# Improving Cycling Performance: Transcranial Direct Current Stimulation Increases Time to Exhaustion in Cycling

**DOI:** 10.1371/journal.pone.0144916

**Published:** 2015-12-16

**Authors:** Marcelo Vitor-Costa, Nilo Massaru Okuno, Henrique Bortolotti, Maurizio Bertollo, Paulo Sergio Boggio, Felipe Fregni, Leandro Ricardo Altimari

**Affiliations:** 1 Universidade Estadual de Londrina. Group of Study and Research in Neuromuscular System and Exercise. Londrina, PR, Brazil; 2 Universidade Estadual de Ponta Grossa. Study Group of Physiological Responses and Adaptations to Exercise. Ponta Grossa, PR, Brazil; 3 Behavioral Imaging and Neural Dynamics Center, Department of Medicine and Aging Sciences, University G. d’Annunzio, Chieti, Italy; 4 Cognitive Neuroscience Laboratory and Developmental Disorders Program, Center for Health and Biological Sciences, Mackenzie Presbyterian University, Sao Paulo, Brazil; 5 Spaulding Neuromodulation Center, Spaulding Rehabilitation Hospital and Massachusetts General Hospital, Harvard Medical School, Boston, Massachusetts, United States of America; University Medical Center Goettingen, GERMANY

## Abstract

The central nervous system seems to have an important role in fatigue and exercise tolerance. Novel noninvasive techniques of neuromodulation can provide insights on the relationship between brain function and exercise performance. The purpose of this study was to determine the effects of transcranial direct current stimulation (tDCS) on physical performance and physiological and perceptual variables with regard to fatigue and exercise tolerance. Eleven physically active subjects participated in an incremental test on a cycle simulator to define peak power output. During 3 visits, the subjects experienced 3 stimulation conditions (anodal, cathodal, or sham tDCS—with an interval of at least 48 h between conditions) in a randomized, counterbalanced order to measure the effects of tDCS on time to exhaustion at 80% of peak power. Stimulation was administered before each test over 13 min at a current intensity of 2.0 mA. In each session, the Brunel Mood State questionnaire was given twice: after stimulation and after the time-to-exhaustion test. Further, during the tests, the electromyographic activity of the vastus lateralis and rectus femoris muscles, perceived exertion, and heart rate were recorded. RM-ANOVA showed that the subjects performed better during anodal primary motor cortex stimulation (491 ± 100 s) compared with cathodal stimulation (443 ± 11 s) and sham (407 ± 69 s). No significant difference was observed between the cathodal and sham conditions. The effect sizes confirmed the greater effect of anodal M1 tDCS (anodal x cathodal = 0.47; anodal x sham = 0.77; and cathodal x sham = 0.29). Magnitude-based inference suggested the anodal condition to be positive versus the cathodal and sham conditions. There were no differences among the three stimulation conditions in RPE (p = 0.07) or heart rate (p = 0.73). However, as hypothesized, RM- ANOVA revealed a main effect of time for the two variables (RPE and HR: p < 0.001). EMG activity also did not differ during the test accross the different conditions. We conclude that anodal tDCS increases exercise tolerance in a cycling-based, constant-load exercise test, performed at 80% of peak power. Performance was enhanced in the absence of changes in physiological and perceptual variables.

## Introduction

Among the many factors that influence fatigue and exercise tolerance, central nervous system related factors seem to play a signfiicant role. As early as 1890, Angelo Mosso provided preliminary evidence on the effect of cognitive activity on fatigue [1]. Recently, individuals who perform heavy cognitive tasks before physical exercise tasks have been demonstrated to tolerate less exercise and have a higher initial rating of perceived exertion (RPE) compared with exercise in a control situation [2]. Further, a separate study, that investigated the physiology of large muscles, demonstrated that fatigue is related to changes in intracortical excitability (as indexed by intracortical facilation and evidenced by the number of pull-ups [3]); thus, providing additional evidence supporting that cortical targets may play a significant role in the intensity and onset of fatigue [4].

In this context, the use of neuromodulation techniques that modulate the function of the cerebral cortex might increase our understanding of the factors that govern exercise performance. Transcranial direct current stimulation (tDCS) is a noninvasive neuromodulation technique that delivers continuous, low-intensity electrical current, causing significant changes of cortical excitability [5]. The effects of tDCS depend on the polarity that is applied—anodal stimulation enhanced cortical excitability, whereas cathodal stimulation is inhibitory [6,7].

Initial evidence has supported the implementation of tDCS in studies on fatigue, exercise tolerance, and recovery between training sessions [8,9,10]. Cogiamanian et al. [8] reported that the application of anodal tDCS to the primary motor cortex for 10 min after an exercise session increased cortical excitability and time to exhaustion in a second session of isometric elbow flexion exercise.

Nevertheless, most existing studies have used isometric exercise for a small muscle mass [9,10,11]. Only one study has used a cycling exercise, in which anodal tDCS altered parasympathetic modulation, perception of effort, and performance in an incremental exercise test to exhaustion [12]. However, this group did not measure physiological variables to determine the effects of tDCS on performance. The time-to-exhaustion test is a good introductory protocol, because it is sensitive and is recommended in the early stages of tests with new interventions that are designed to boost athletic performance [13].

The objective of this study was to determine the effects of tDCS on time to exhaustion and physiological responses during high-intensity constant-load exercise and on mood before and after exercise. Based on earlier findings, we hypothesized that: a) anodal tDCS would increase exercise tolerance (ie, time to exhaustion), irrespective of changes in heart rate and electromyographic (EMG) activity, whereas cathodal tDCS would fail to improve, or worsen, performance; and b) anodal tDCS conditions would moderate and delay the increase in RPE over time, explaining the improvement in performance.

## Material and Methods

### Subjects

Participants were deemed eligible using the following criteria: (1) age between 18 and 30 years; (2) males (3) no diagnosis of neurological, or psychiatric disorders; (4) no drug or alcohol abuse; (5) not enrolled in another trial involving weight training; (6) being active (practice physical activities at least three times a week for at least six months). Fifteen physical active subjects met our inclusion criteria and agreed to participate initially in the study. Two subjects dropped out for personal reasons, one subject dropped out without providing any reason, and one because he was afraid to receive the stimulation. Thus, the sample consisted of 11 male subjects aged 21–31 years (M = 26 SD = 4), see Table 1. All subjects received detailed information about the proposal of the study and the procedures, and signed the informed consent form. The study was approved by the Ethics Committee of the State University of Londrina (Universidade Estadual de Londrina; Permit No. 19779/2011).

**Table 1:** Anthropometric characteristics and performance of the subjects studied in the incremental test (N = 11).

	Mean ± standard deviation
Weight (kg)	77 ± 15
Height (cm)	177 ± 3
Peak power (W)	257±35
80% of peak power (W)	205 ± 28
Maximum heart rate (bpm)	187± 10

### Study design

This study was a single blinded, randomized, placebo-controlled, crossover study with a repeated measures design. First, the subjects were seen at the laboratory for consenting and scheduling. The order of stimulation conditions were then randomized using the following site: http://www.randomization.com. All subjects received all stimulation conditions in a counterbalanced order.

The subjects visited the laboratory four times (one pre-experimental and three experimental sessions). In the pre-experimental session, the subjects performed an incremental test to determine peak power. In the subsequent visits, all procedures were the same, except for the stimulation conditions, which were previously randomized.

First, the transmitter strap of the heart rate monitor was fixed to the subject’s chest and the subject was asked to sit comfortably in a chair. The electrodes for tDCS were maintained on the subject’s head with a rubber band. After the beginning of tDCS, the skin of the thigh was prepared and electrodes were placed for EMG recording. At the end of tDCS, the subjects answered a questionnaire for the assessment of mood state. Next, the subjects walked to a cycle simulator and started a standard warm-up, pedalling at 40% of peak power for 4 min, followed immediately by a test for EMG normalization. Finally, after 5 min of rest, the subjects performed a constant-load test (time to exhaustion). The subjects were asked to pedal at 60 to 90 rpm until voluntary exhaustion against a resistance corresponding to 80% of the peak power obtained in the pre-experimental test (incremental test). Feedback about cycling cadence was provided and displayed on a screen positioned in front of the subject. The criterion adopted for interruption of the test was a decrease in the required pedalling cadence without recovery for more than 5 s. Immediately after the end of exercise, the subjects again filled the mood state questionnaire.

### Incremental test

For the incremental test, the subjects warmed up for 2 min at 100 W. Next, the workload was increased by 50 W at intervals of 2 min until voluntary exhaustion or inability to maintain the established minimum cadence (60 rpm) for more than 5 s. The power achieved in the last complete stage added to the product of the percent time spent in the stage of exhaustion by the standard increment (50 W) was defined as the peak power output. The position of the cycle ergometer was adjusted individually and the specifications were recorded so that it could be reproduced in the subsequent visits.

### Constant-load Test

During the following three visits, the subjects performed a constant-load test in which they were asked to pedal until exhaustion at 80% of the load achieved in the incremental test. The test was interrupted due to voluntary exhaustion or when the subjects could no longer maintain the pedalling cadence (60 to 90 rpm) for more than 5 s. The Velotron Dynafit Pro™ cycle simulator (RacerMate®, Seattle, WA, USA), with a capacity of up to 1500 W, was used for all physical tests.

### tDCS procedures

The electrical current was applied with a portable apparatus consisting of four main components: electrodes (anode and cathode with an area of 35 or 36 cm2), ammeter (measures the intensity of the electrical current), potentiometer (component that permits manipulation of current intensity), and three batteries (9 V) to generate the current. The electrodes were wrapped in a saline (150 mM NaCl)-soaked sponge. The equipment used was built by an electronic engineer and has been previously approved by our Local Ethics Committee.

**Fig 1:**
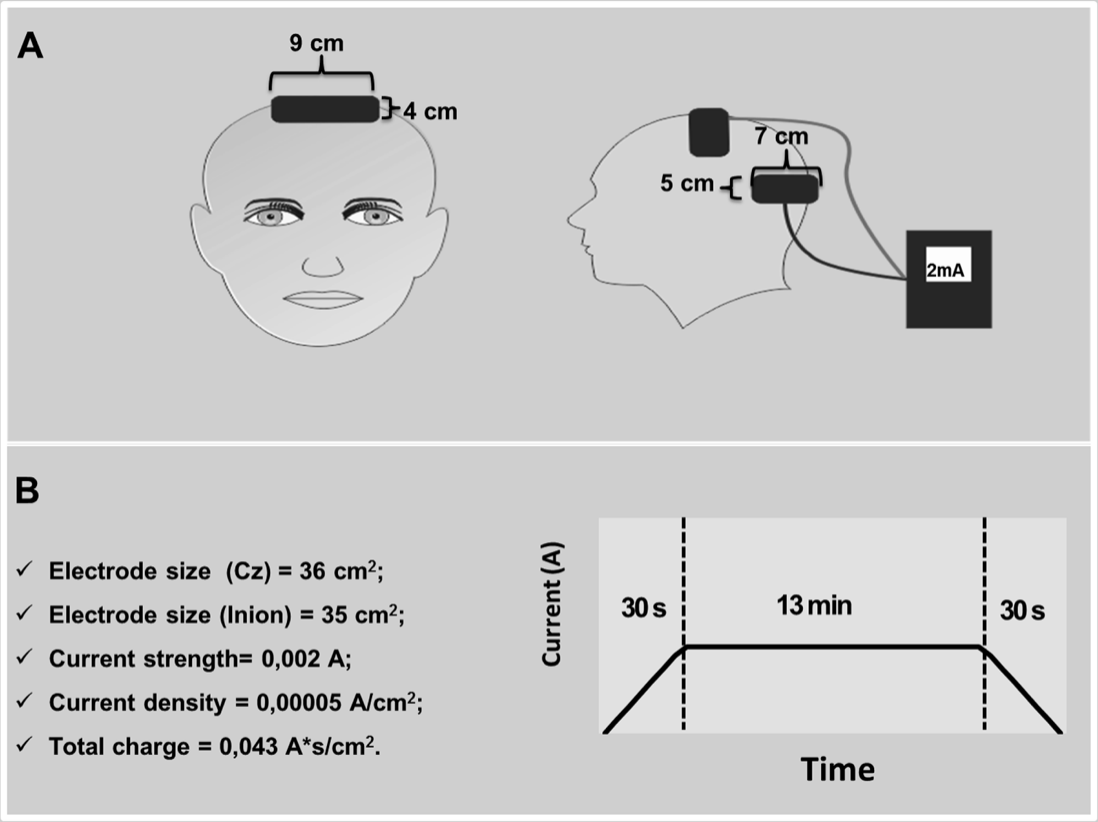
(A) Schematic illustration of the position of the electrodes during stimulation. (B) Stimulation parameters and an example of current ramp up and ramp down at the beginning and end of tDCS.

The EEG 10–20 international system [14] was used for electrode positioning. The goal of electrode size and placement was to induce current in both left and right M1 simultaneously [15]. Both brain hemispheres were stimulated, because in the chosen exercise, the subjects cycled with both lower limbs. Therefore, the centre of one electrode (9 x 4 cm) was placed in Cz region (thus 4.5 cm of each side of the primary motor cortex). Another electrode (7 x 5 cm) was placed on the occipital protuberance. The subjects received 2-mA stimuli over a period of 13 min (see Fig 1).

Despite reports of some side effects, tDCS is considered to be a safe technique [16]. The most common side effects are tingling and itching under the electrodes. Nausea, insomnia and headache are less frequently reported. It should be noted that these side effects have also been observed in sham conditions.

### Surface electromyography

A torque-velocity test was used to normalize EMG activity during the time-to-exhaustion test [17]. After warm-up in the experimental session, the subject performed an 8-s maximum sprint. The load imposed during the test was 7.5% of the body weight of each volunteer. The participants were asked to remain seated throughout the test. The EMG activity of each muscle was analysed between the second and eighth second of the sprint and the highest peak of the amplitude found, expressed as RMS, was used as the normalization factor.

EMG activity was measured continuously during the test in the three experimental sessions. Data were collected using an 8-channel EMG system (TeleMyo 2400TG2, Noraxon, Inc., USA). The sampling frequency of the electromyographic recordings was 2000 Hz. The common mode rejection ratio was ≤ 95 dB.

The following muscles were analysed: vastus lateralis (VL), and rectus femoris (RF). Standards of the International Society of Electrophysiological Kinesiology (ISEK) were followed for the EMG data collection. The active electrodes (TeleMyo 2400, Noraxon, Inc., USA), with an interelectrode (centre to centre) distance of 2 cm, were placed after shaving of the site, asepsis with alcohol and curettage to reduce impedance of the skin. The reference electrode was placed on the olecranon.

The electrodes were located on the muscles analysed placed according to Surface Electromyography for the Non-Invasive Assessment of Muscles (SENIAM) [18] recommendations. Electrodes on the VL muscle were placed at two-thirds of the distance between the anterior superior iliac spine and lateral border of the patella. Electrodes on the RF were placed half the distance between the iliac crest and superior border of the patella.

Root mean squares (RMS) and median frequencies (mF) obtained at intervals of 5 s were the indices used to evaluate the activity of the RF and VL muscles [19]. The data were processed with the MatLab 7.0 software (MathWorks®, South Natick, MA, USA). A band pass filter from 20 to 500 Hz was used to filter raw data before analysis.

### Rating of perceived exertion (RPE) and Mood state (BRUMS)

All subjects self-rated overall perceived exertion using the Borg scale (6–20 points) [20] every minute of the time-to-exhaustion test. The subjects followed the instructions recommended by Borg to avoid any confusion among the perception of effort, discomfort or any other unpleasant sensation.

The volunteers filled out the Brazilian version of the Brunel Mood Scale (BRUMS) immediately before and after the experimental test session [21]. The questionnaire consists of 24 items divided into six domains: confusion, anger, depression, fatigue, tension, and vigour. The data were analysed based on the scores obtained for each domain before the experimental test and were also normalized by subtracting the score obtained at the end of the test from the score obtained before the tests.

### Heart rate

Heart rate was measured continuously during all tests with an RS800 heart rate monitor (Polar®, Oy, Finland), with beat-to-beat recordings using the Polar Precision Performance software (Oy, Finland) for subsequent analysis. Heart rate was analysed using the mean of 5 s obtained at the end of each minute of the test.

### Statistical analysis

For the sample size calculation, given this was an exploratory study we used a moderate effect size. If the results were not significant that would indicate that there would be no differences between the groups or the difference would be so small that would not be relevant. As we used ANOVA as the main method of analysis, a moderate effect size would be a partial η^2^ = 0.06. The sample size was calculated using G*Power 3.1.7 considering power of 0.80, a correlation among the measurements of 0.85, nonsphericity correction ϵ = 0.75 and as aforementioned a partial η^2^ of 0.6. Using these parameters, sample size calculation resulted in a sample size of 11 subjects.

The assumptions of Normality and sphericity of the data were checked using the Shapiro-Wilk test and the Mauchly test and all the data showed normal distribution and sphericity. Repeated measures analysis of variance (RM-ANOVA) followed by the Bonferroni post hoc test were used for comparison of time to exhaustion in the constant-load test and of BRUMS scores between the different stimulation conditions. Two-way ANOVA for repeated measures was used for analysis of EMG, HR and RPE response, using the stimulation conditions and time as main factors. Alpha level was set at p<0.05. In addition, magnitude-based inference was applied to evaluate the time to exhaustion [22]. For this purpose, the percent chance was determined that the changes, obtained by logarithmic transformation, observed after the application of anodal or cathodal tDCS had a positive, trivial or negative effect compared to sham tDCS. The probability of the effects found was analysed as follows: <1% almost certainly not positive/inconclusive/negative; 1–5% very unlikely positive/inconclusive/negative; 5–25% unlikely positive/inconclusive/negative; 25–75% possibly positive/inconclusive/negative; 75–95% likely positive/inconclusive/negative; 95–99% very likely positive/inconclusive/negative, and >99% almost certainly positive/irrelevant/negative. If the negative and positive values presented results > 10%, the inference was considered inconclusive. The spreadsheets [23] available at http://www.sportsci.org/resource/stats/index.html were used for these analyses. In addition, the effect size (Cohen d) [24] was calculated for performance in the time-to-exhaustion test and interpreted using the recommendations proposed by Hopkins (3): < 0.2 trivial; 0.2–< 0.6 small; 0.6–< 1.2 moderate; 1.2–< 2.0 large; 2.0–< 4.0 very large; 4.0 nearly perfect (http://www.sportsci.org/resource/stats). Our spreadsheet including raw data is available at the following link: http://datadryad.org/review?doi=doi:10.5061/dryad.30p8j.

## Results and Discussion

The intervention was well tolerated by all subjects. Only one subject reported weak headache after anodal stimulation.

**Fig 2:**
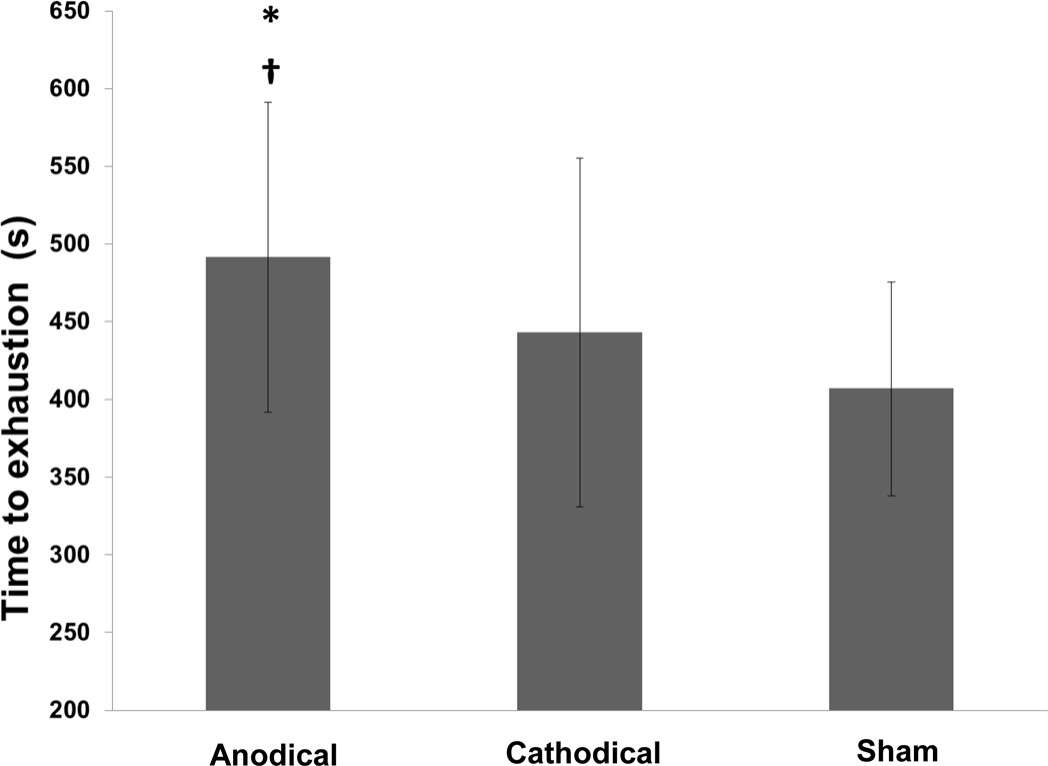
Time to exhaustion under the different experimental conditions. * Significant difference compared to the sham condition (p = 0.02); † significant difference compared to the cathodal condition (p = 0.008).

**Fig 3:**
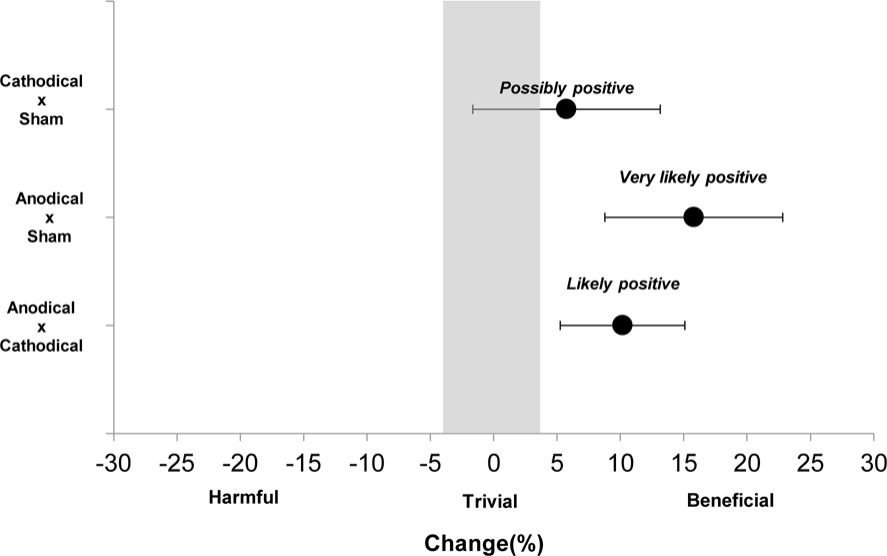
Percent change between the different stimulation conditions. The qualitative inferences were: cathodal x sham = 68 beneficial/31 trivial/2 harmful; anodal x sham = 99 beneficial/1 trivial/0 harmful; anodal x cathodal = 96.1 beneficial/3.8 trivial/0.03 harmful.

A one-way repeated measure ANOVA was conducted to compare the effect of stimulation type (anodal, cathodal and sham) on time to exhaustion. There was a significant effect of stimulation type, Wilks’ Lambda = 0.33, F (2,9) = 9.14, p = 0.007. The Bonferroni corrected post hoc tests (we also performed Cohen d effect size to further evaluate the effect of our experimental intervention) found the time to exhaustion was significantly larger in subjects receiving anodal stimulation as compared to those receiving cathodal (Cohen d = 0.47, small, p = 0.020) and sham (Cohen d = 0.7, moderate, p = 0.008) stimulation (Fig 2). However, no significant difference (Cohen d = 0.29, small, p = 0.496) was observed between cathodal and sham stimulation (Fig 2). Qualitative analysis also showed a greater magnitude of change in the test for subjects receiving anodal stimulation compared to the other conditions (Fig 3).

Additionally, the post hoc analysis to compute achieved power on time to exhaustion results based on the parameters generated by ANOVA (partial η^2^ = 0.65, nonsphericity correction ϵ = 0.85) presented value equal to 1.

**Fig 4:**
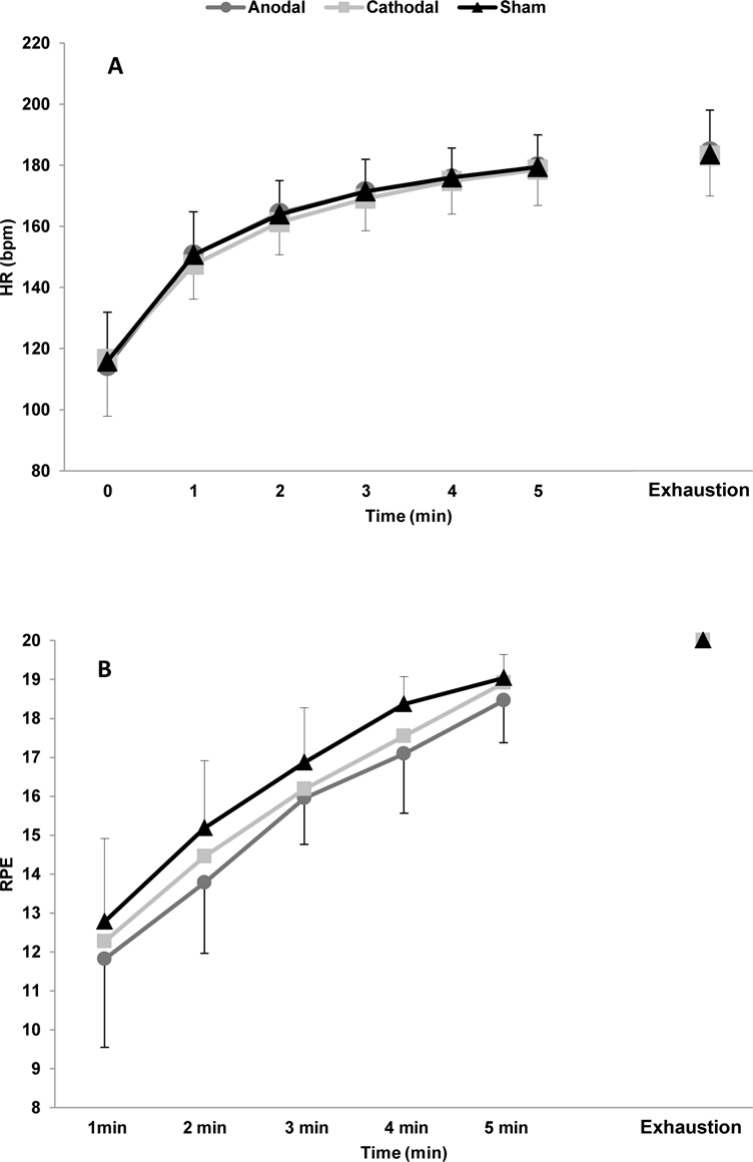
Subjective rating of perceived exertion (RPE) (A) and heart rate (HR) (B) along the time-to-exhaustion test under the three experimental conditions.

There were no differences among the three stimulation conditions in RPE (Wilks’ Lambda = 0.55, F (2,9) = 3.730, p = 0.07) (Fig 4A) or heart rate (Wilks’ Lambda = 0.92, F (2,8) = 0.328, p = 0.73) (Fig 4B). However, ANOVA revealed only a main effect of time for the two variables (RPE: Wilks’ Lambda = 0.03, F (5,6) = 36.115; HR: Wilks’ Lambda = 0.005, F (6,4) = 124.113, p < 0.001), as expected.

**Fig 5:**
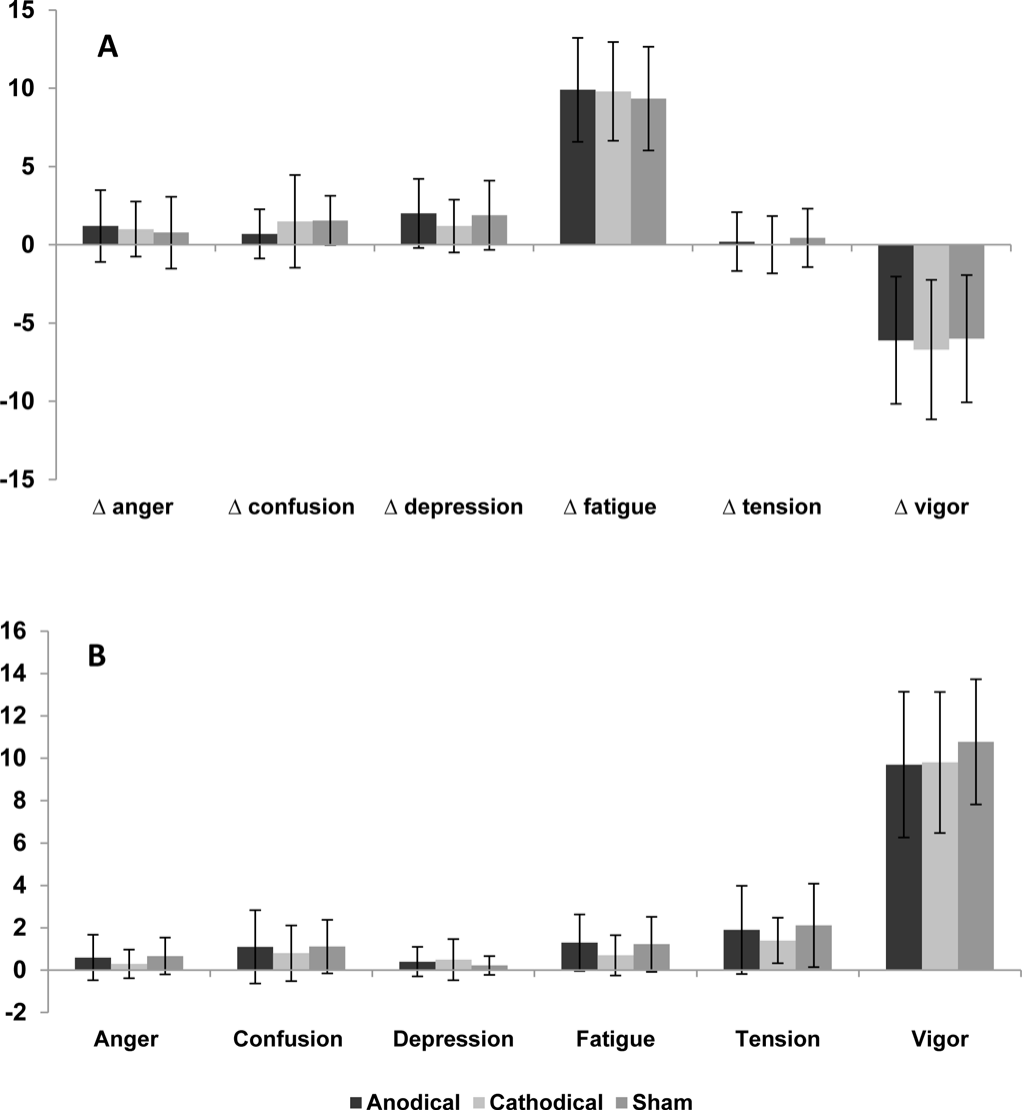
Mood state reported as delta value (A) and BRUMS score before exercise (B).

Furthermore, no significant differences were observed in any of the domains of the BRUMS scales. Fig 5 shows the results of each domain of mood states: anger (Wilks’ Lambda = 0.64, F (2,7) = 1.90, p = 0.218), confusion (Wilks’ Lambda = 0.93, F (2,7) = 0.25, p = 0.785), depression (Wilks’ Lambda = 0.73, F (2,7) = 1.25, p = 0.341), fatigue (Wilks’ Lambda = 0.67, F (2,7) = 1.65, p = 0.258), tension (Wilks’ Lambda = 0.63, F (2,7) = 2.00, p = 0.205), and vigour (Wilks’ Lambda = 0.92, F (2,7) = 0.92, p = 0.748). Analysis of delta variation in mood states also revealed no significant differences, irrespective of the domain analysed: anger (F (2,7) = 1.55; p = 0.242), confusion (F(2,7) = 0.82; p = 0.458), depression (F(2,7) = 2.8; p = 0.09), fatigue (F (2,7) = 0.60; p = 0.561), tension (F(2,7) = 0.17; p = 0.840), and vigour (F(2,7) = 1.24; p = 0.315).

**Fig 6:**
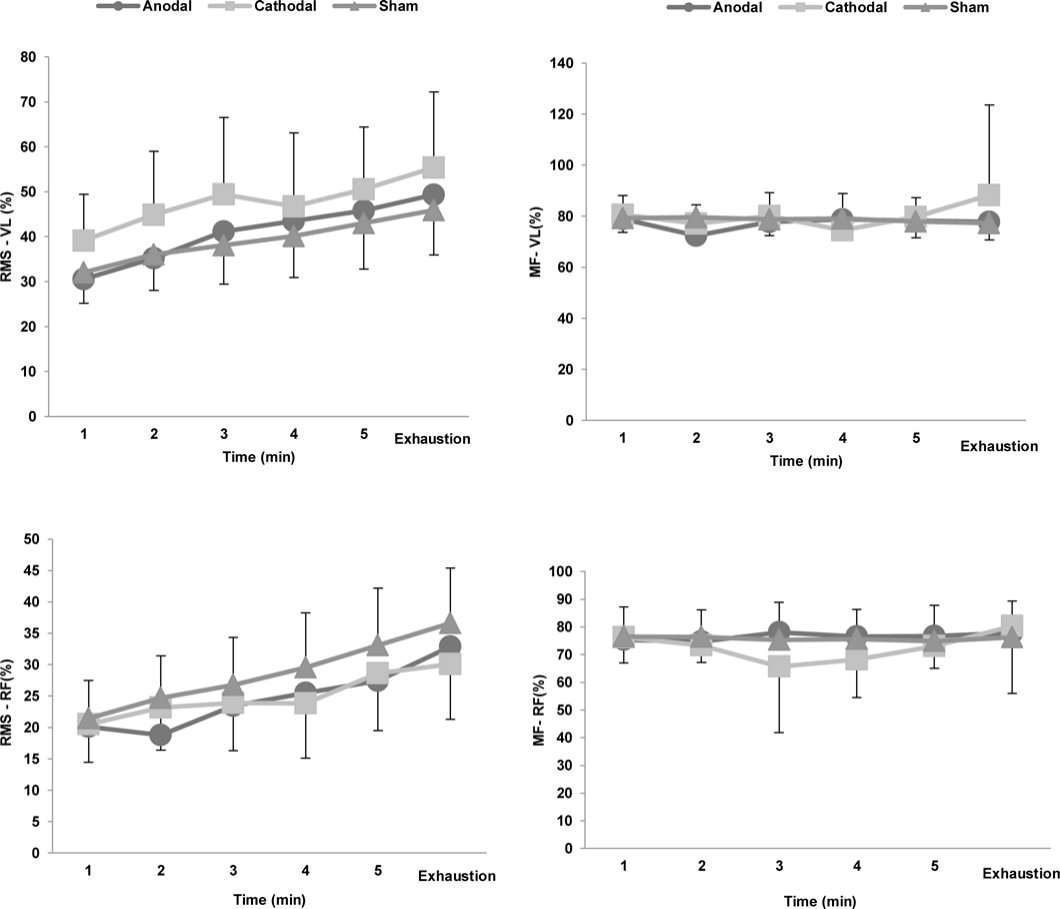
Electromyography responses of the vastus lateralis (VL) and rectus femoris (RF) muscles during the time-to-exhaustion test. Normalized by the values obtained in the torque-velocity test. In the time domain, the results are reported as root mean square (RMS) and in the frequency domain as median frequency (MF).

The peak power achieved in the torque-velocity test did not differ between conditions (anodal = 836 ± 207 W, cathodal = 864 ± 294 W, and sham = 840 ± 246 W; F = 0.215; p = 0.809). EMG activity also did not differ during the test among the different conditions (Fig 6).

The main finding of our study is the increase of exercise tolerance in the time-to-exhaustion test after anodal tDCS, irrespective of differences in physiological variables (heart rate, EMG), consistent with our initial hypothesis. However, in contrast to our expectations, perceptual and affective variables (RPE, BRUMS) did not differ between experimental conditions.

Specifically, our results are consistent with Cogiamanian et al. [8], who reported an increase in exercise tolerance after application of anodal stimulation for upper limb isometric exercise, without alterations in EMG indices. Nonetheless, application of a cathodal current did not affect performance. Despite their similarities, these studies differed with regard to the time and configuration of the electrodes during tDCS application—parameters that can influence the changes in cortical excitability that are induced by tDCS [6,16].

In this study, the configuration of the electrodes was selected to modulate motor cortex excitability and facilitation in both hemispheres [15], because the exercise protocol involves a large muscle mass in both legs. The reference electrode in our study was not placed at Fp2 or Fp3, as in most studies. Instead, we placed it in the occipital cortex to prevent alterations in the prefrontal regions of the cerebral cortex, which are related to the perception of effort [25].

Another methodological strength of our study was our decision to fix the constant-load test at 80% of peak power in a previous incremental test. This method shows good sensitivity when the objective is to evaluate factors that alter endurance exercise performance and allows us to analyse physiological responses in a controlled manner [13]. The heart rate (Fig 4) and EMG (Fig 6) responses behaved similarly over the 3 experimental sessions, whereas exercise tolerance was higher under the anodal tDCS condition.

Similarly, to our study, in which tDCS increased the time to exhaustion, other groups demonstrated that tDCS improve performances in other tasks by enhancing muscle strength. For instance, Tanaka et al. [9] observed that anodal tDCS increases pinch force during and 30 min after stimulation in healthy subjects. Later, in patients with stroke, Tanaka et al. [10] noted improvements in knee extensor strength of the paretic leg during application of anodal tDCS, but no difference was seen after 30 min.

In contrast to our initial hypothesis, rating of perceived exertion (RPE) was unaltered between experimental conditions. It is possible that the area of stimulation (Cz) does not interfere with perceptual responses. The perception of effort is related to activity in the insular cortex, anterior cingulate cortex, thalamus, and frontal operculum. Further, an increase in RPE correlates with an increase in central motor commands (movement-related cortical potential), as measured by electroencephalography at the Cz [26]. This relationship should be interpreted with caution, because in hypnosis experiments, in which the subjects were asked to imagine themselves performing an exercise, RPE rose, regardless of motor command [27,28]. It is also possible that subjects adjusted their level of effort according to time to exhaustion.

A study on direct electric stimulation in patients who were undergoing surgery reported that when the electrical stimulus was applied to the parietal cortex, the subjects believed that they had performed movements. However, no EMG activity or movement was observed. Conversely, stimulation of the premotor cortex triggered mouth and contralateral limb movements, which the patients denied that they made [29]. These data suggest that motor commands are not necessary for the formation of movement perception or the perception of effort.

In our study, no difference in EMG responses or heart rate was observed between the 3 experimental conditions. This finding was expected, because physiological variables are well controlled in time-to-exhaustion tests, as in this study [13], in which the load was constant throughout the test and the same load was used across experimental conditions. Our results agree with those of Marcora et al. [2], who subjected participants to mental fatigue before a time-to-exhaustion test. Although exercise tolerance declined after mental fatigue, no difference in physiological variables was observed compared with the control condition.

We suggest that the mechanisms of longer exercise tolerance that is mediated by anodal tDCS are related to an increase in intracortical facilitation and motor cortex excitability [30]. Thus TDCS allows to cycle for longer periods, because intracortical facilitation correlates with total workload when doing pull-ups [3]. To this end, a facilitation system for the motor cortex during exercise until exhaustion has been proposed [31,32].

In particular, Tanaka and Watanabe [32] developed a neural circuit for the action of this facilitatory pathway. First, sensory input from the peripheral system to the primary motor cortex reduces motor output (supraspinal fatigue), and a neural pathway that interconnects the spinal cord, thalamus, secondary somatosensory cortex, medial insular cortex, posterior cingulate cortex, anterior cingulate cortex, premotor area, supplementary motor area, and primary motor cortex constitutes the inhibition system. Then, a facilitation system increases motor output from the primary motor cortex to overcome the existing supraspinal fatigue. A re-entrant neural circuit that bridges the limbic system, basal ganglia, thalamus, orbitofrontal cortex, prefrontal cortex, anterior cingulate cortex, premotor area, supplementary motor area, and primary motor area represents the facilitation system. Motivational input to this system enhances supplementary motor area activity, and subsequently, motor cortex enhances motor output to the peripheral system.

Thus, the output (exit of information from the motor cortex to the corticospinal pathways and, consequently, motoneurons) from the primary motor cortex is regulated primarily by the balance between inhibition and facilitation, leading us to speculate that anodal tDCS at the Cz has a facilitatory effect for longer periods, increasing exercise tolerance.

Nevertheless, we agree that the balance between inhibitory and excitatory information in the primary motor cortex determines the amount of motor output and, consequently, the end of exercise. In addition, tDCS alters corticospinal excitability, and thus, the effects might not be restricted to changes in intracortical excitability but might extend, for instance, to distant areas of the motor corticospinal tract.

We cannot directly confirm that the changes in excitability and intracortical facilitation are responsible for altering the performance observed in our study. However, some authors showed that changes in excitability and intracortical facilitation are responsible for changing the performance of locomotor muscles [33, 34]. In particular, Sidhu et al. [33] reported an impairment in maximal voluntary activation after exhaustive exercise in these muscles, suggesting that fatigue can impair the neural drive for locomotor muscles.

Therefore, intracortical facilitation probably occurs when time to exhaustion increases, but further studies are necessary to confirm this hypothesis. Furthermore, the tDCS montage can influence other brain areas related to performance, such as parietal cortex or occipital. A more specific stimulation should be applied to investigate each area separately.

## Conclusion

Our results suggest that anodal tDCS increases exercise tolerance in a cycling-based, constant-load exercise test, performed at 80% of peak power. Performance was enhanced in the absence of changes in physiological variables, such as heart rate and EMG, and perceptual variables, such as RPE, during the test. Moreover, anodal tDCS did not alter the profile of mood states before or after the time-to-exhaustion test versus cathodal and sham conditions. These results, if confirmed in time-trial protocols, will advance the theoretical and applied use of tDCS in improving athletic performance and its examination in future studies to better understand the neurophysiological mechanisms of exercise tolerance and fatigue.

Additional studies should be conducted to confirm our results—for instance, experiments that determine whether tDCS alters performance in pacing exercises, such as time-trial exercise, and studies that measure the effects of tDCS as recovery technique between exercise sessions. In addition, whether tDCS can be used as a tool for recovery in athletes with signs and symptoms of non-functional overreaching and overtraining should be examined. tDCS might be applied not only to cycling activities but also to tasks that require the acquisition and retention of new motor skills and to high-precision sports, such as shooting and archery.
